# Post-Mortem Immunohistochemical Evidence of β2-Adrenergic Receptor Expression in the Adrenal Gland

**DOI:** 10.3390/ijms20123065

**Published:** 2019-06-23

**Authors:** Elvira Ventura Spagnolo, Cristina Mondello, Luigi Cardia, Letteria Minutoli, Domenico Puzzolo, Alessio Asmundo, Vincenzo Macaione, Angela Alibrandi, Consuelo Malta, Gennaro Baldino, Antonio Micali

**Affiliations:** 1 Legal Medicine Section, Department for Health Promotion and Mother-Child Care, University of Palermo, Via del Vespro, 129, 90127 Palermo, Italy; gennarobld@hotmail.it; 2 Department of Biomedical and Dental Sciences and Morphofunctional Imaging, University of Messina, via Consolare Valeria, 1, 98125 Messina, Italy; puzzolo@unime.it (D.P.); aasmundo@unime.it (A.A.); consuelo_malta@hotmail.it (C.M.); amicali@unime.it (A.M.); 3 Department of Human Pathology of Adult and Childhood “Gaetano Barresi”, University of Messina, Via Consolare Valeria, 98125 Gazzi, Italy; luigicardia1@gmail.com; 4 Department of Clinical and Experimental Medicine, University of Messina, via Consolare Valeria, 1, 98125 Messina, Italy; lminutoli@unime.it (L.M.); vmacaione@unime.it (V.M.); 5 Department of Economics, Unit of Statistical and Mathematical Sciences, University of Messina, Via dei Verdi 75, 98122 Messina, Italy; aalibrandi@unime.it

**Keywords:** adrenal gland, β2-adrenergic receptors, immunohistochemistry, post-mortem analysis

## Abstract

The evidence from post-mortem biochemical studies conducted on cortisol and catecholamines suggest that analysis of the adrenal gland could provide useful information about its role in human pathophysiology and the stress response. Authors designed an immunohistochemical study on the expression of the adrenal β2-adrenergic receptor (β2-AR), a receptor with high-affinity for catecholamines, with the aim to show which zones it is expressed in and how its expression differs in relation to the cause of death. The immunohistochemical study was performed on adrenal glands obtained from 48 forensic autopsies of subjects that died as a result of different pathogenic mechanisms using a mouse monoclonal β2-AR antibody. The results show that immunoreactivity for β2-AR was observed in all adrenal zones. Furthermore, immunoreactivity for β2-AR has shown variation in the localization and intensity of different patterns in relation to the original cause of death. To the best of our knowledge, this is the first study that demonstrates β2-AR expression in the human cortex and provides suggestions on the possible involvement of β2-AR in human cortex hormonal stimulation. In conclusion, the authors provide a possible explanation for the observed differences in expression in relation to the cause of death.

## 1. Introduction

The adrenal response to stress involves the activation of the sympathoadrenal (SA) system and the hypothalamic–pituitary–adrenocortical (HPA) axis; in this way, homeostasis in emergencies such as “fight or flight” situations is maintained [1]. Several studies were performed to explain the activities of these mechanisms and it was demonstrated that the stress response activates the sympathetic nervous system (SNS) and the subsequent release of catecholamines from the sympathetic nervous system terminals and the adrenal glands [2]. However, scientific evidence has shown that both the SNS and the adrenomedullary system can respond differentially to stress in relation to the type and the intensity of the stressor and to the perception and interpretation of the stress situation [3]. In this setting, the adrenomedullary secretion was also associated with the concurrent activation of the HPA axis [4].

In the forensic field, many researchers analyzed the individual differences in the stress response during the death process, mostly investigating both catecholamine and glucocorticosteroid levels, using a biochemical approach [5,6,7,8] and immunohistochemistry [9]. Nevertheless, catecholamines have been considered as an unstable marker in the post-mortem investigation of the causes or the process of death [5]. Moreover, very few studies in forensic pathology have been carried on the adrenal gland, despite it being one of the main organs involved in the stress response, releasing corticosteroids and catecholamines [10,11]. Catecholamines are able to up-regulate the expression of membrane-bound proteins known as adrenergic receptors (ARs), and the β2-AR pathway, in many organs, either in normal [12] or in pathological conditions [13,14], suggesting that the analysis of the molecular change of such receptors could provide information on specific circumstances related to stressful events.

ARs are cell membrane receptors included in the seven-transmembrane-spanning G-protein coupled receptor (GPCR) superfamily, formed by membrane proteins triggered by extracellular ligands such as small organic molecules, peptides, and neurotransmitters [15]. Structurally, GPCRs are formed by seven hydrophobic transmembrane segments which cross the plasma membrane with an extracellular amino terminus and an intracellular carboxyl-terminus. Intracellularly, GPCRs interact with G proteins, which induce the stimulation of numerous targets [16,17,18]. ARs mediate the action of the sympathetic neurotransmitter noradrenaline and the hormone adrenaline, even if some are of higher affinity to the former molecule [19].

According to their pharmacological response, ARs were subdivided into α and β types: these latter types were further divided into three families called β1, β2 and β3 on the basis of molecular classification [19,20]. β2-ARs have been described in multiple locations such as the lung, heart, blood vessels, and nasal turbinates [13,15,20]. However, there is little data available on the presence of β2-ARs in the adrenal gland. In isolated adrenal chromaffin cells, the presence of β2-ARs was demonstrated both in rats [21] and in humans [22], suggesting that, at least in vitro, catecholamine release could be modulated by the activation of β2-ARs. Furthermore, in primary cultures of cells from the fasciculata and the reticularis zones of the bovine adrenal gland, the presence of β2-ARs was demonstrated [23].

The purpose of this study was to analyze the adrenal glands obtained from forensic autopsies in order to evaluate the expression of β2-AR in the adrenal glands and to investigate its expression in subjects with different causes of death.

## 2. Results

**Figure 1:**
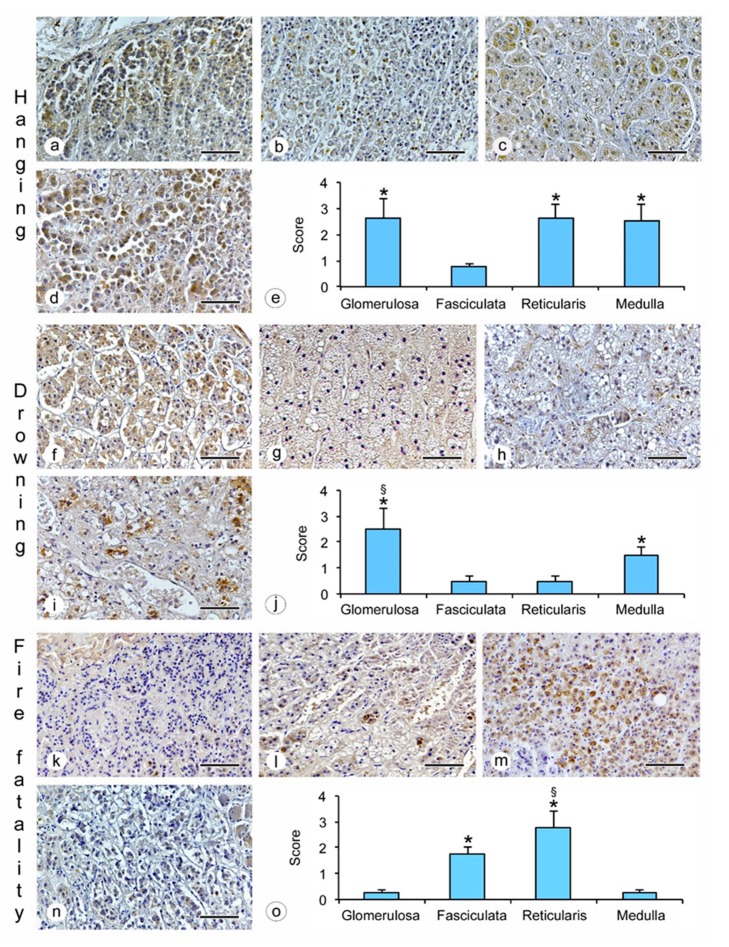
β2-AR expression in cases of hanging, drowning and fire fatality in the different zones of the adrenal gland. **a**,**f**,**k** = glomerulosa zone; **b**,**g**,**l** = fasciculata zone; c,h,m = reticularis zone; **d**,**i**,**n** = medulla; **e** = levels of immunohistochemical expression in the different zones of the adrenal gland of subjects from the hanging group based on the arbitrary score used. * = *p* < 0.05 versus fasciculata zone; **j** = levels of immunohistochemical expression in the different zones of the adrenal gland of subjects from the drowning group based on the arbitrary score used. * = *p* < 0.05 versus glomerulosa zone and medulla, § = *p* < 0.05 versus medulla; **o** = levels of immunohistochemical expression in the different zones of the adrenal gland of subjects from the fire fatality group based on the arbitrary score used. * = *p* < 0.05 versus glomerulosa zone and medulla, § = *p* < 0.05 versus fasciculata zone. (Scale bar = 100 μm).

**Figure 2:**
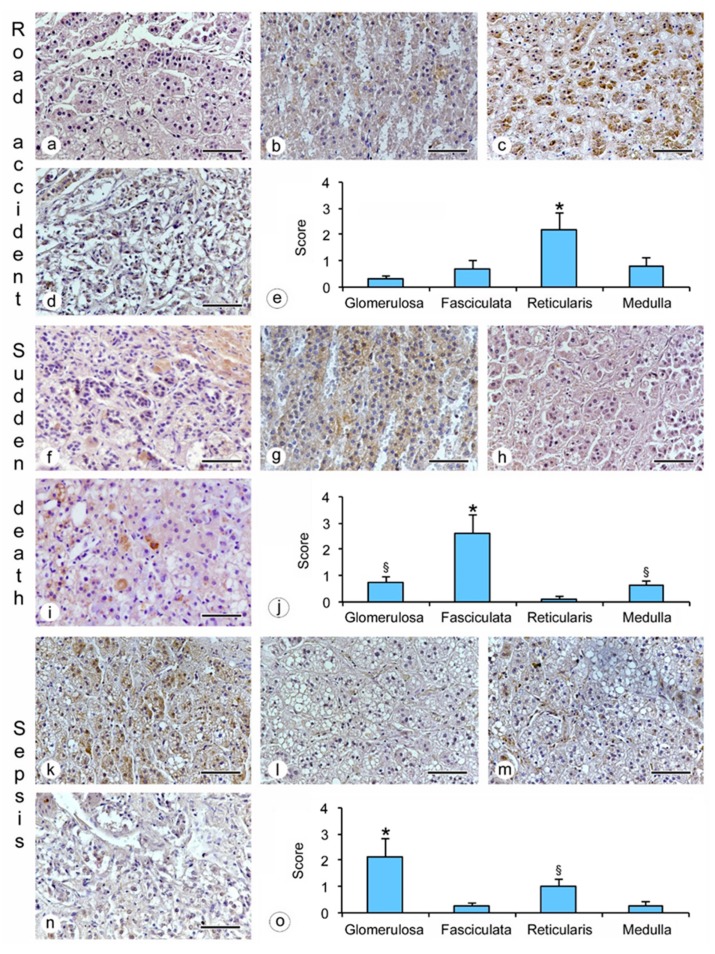
β2-adrenergic receptor (AR) expression in cases of road accidents, sudden death and sepsis in the different zones of the adrenal gland. **a**,**f**,**k** = glomerulosa zone; **b**,**g**,**l** = fasciculata zone; **c**,**h**,**m** = reticularis zone; **d**,**i**,**n** = medulla; **e** = levels of immunohistochemical expression in the different zones of the adrenal gland of subjects from the road accident group based on the arbitrary score used. * = *p* < 0.05 versus glomerulosa and fasciculata zones and medulla; **j** = levels of immunohistochemical expression in the different zones of the adrenal gland of subjects from the sudden death group based on the arbitrary score used. * = *p* < 0.05 versus glomerulosa and reticularis zones and medulla, § = *p* < 0.05 versus reticularis zone; o = levels of immunohistochemical expression in the different zones of the adrenal gland of subjects from the sepsis group based on the arbitrary score used. * = *p* < 0.05 versus fasciculata and reticularis zones and medulla, § = *p* < 0.05 versus fasciculata zone and medulla. Scale bar = 100 μm.

The study revealed an evident immunoreactivity for β2-AR expressed in the human adrenal gland (glomerulosa, fasciculata, reticularis and medulla zones) (Figure 1 and Figure 2).

Moreover, the positive staining varied in localization and intensity in the specimens obtained from subjects included in the six groups examined, as shown in Table 1:

- hanging: the immunostaining of β2-AR was higher in the glomerulosa, reticularis, and medulla (Figure 1a,c,d), while a lower expression was observed in the fasciculata (Figure 1b). The statistical analysis confirmed the significant differences among the zones (*p* < 0.05 versus the fasciculata zone) (Figure 1e);
- drowning: the β2-AR immunostaining was higher in the glomerulosa and the medulla (Figure 1f,i), and significantly lower in the fasciculata and in the reticularis (Figure 1g,h). The statistical analysis also showed significant differences (*p* < 0.05) between the glomerulosa and the medulla (Figure 1j).
- fire fatality: the fasciculata and the reticularis zones (Figure 1l,m) were significantly positive for β2-AR when compared to the other zones (Figure 1k,n). In these cases, a significant difference was also observed between the fasciculata and the reticularis (*p* < 0.05) (Figure 1o);
- road accident: if compared to the other adrenal zones (Figure 2 a,b,d), only the reticularis (Figure 2c) showed evident β2-AR immunostaining, which was also confirmed by the statistical analysis (Figure 2e);
- sudden cardiac death: the glomerulosa and the medulla showed mild β2-AR immunostaining (Figure 2f,i), while the fasciculata (Figure 2g,j) was significantly positive for β2-AR; the reticularis was negative (Figure 2h).
- sepsis: the highest β2-AR immunostaining was observed in the glomerulosa (Figure 2k); the positivity in the reticularis was less evident (Figure 2m), but significantly higher than in the fasciculata and the medulla (Figure 2l,n,o).

**Figure 3:**
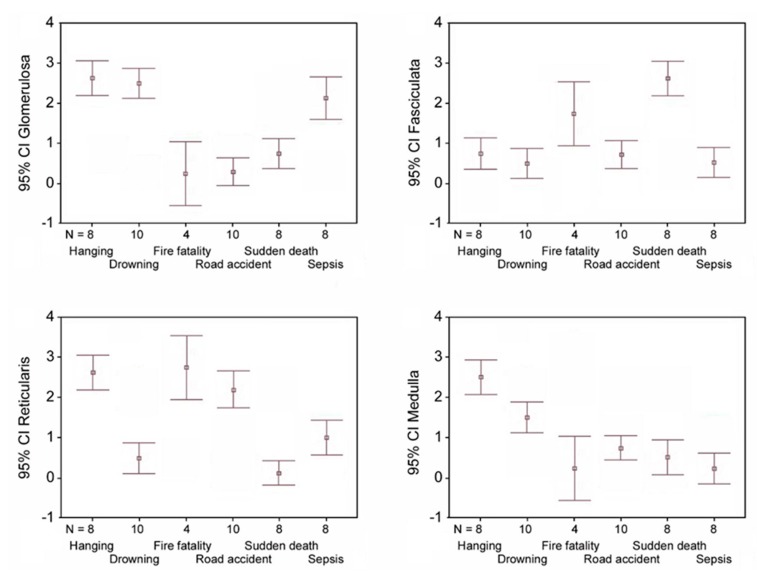
Error bar graphs show mean value and relative 95% confidence interval (CI) of β2-AR expression of each adrenal zone (glomerulosa, fasciculata, reticularis, medulla) in the different causes of death examined.

As a consequence of the above results, the comparison between β2-AR expression in the single zones showed that: (a) the glomerulosa zone has a higher expression in hanging, drowning and sepsis deaths; (b) the fasciculata zone was significantly positive in sudden cardiac death and fire fatalities; (c) the reticularis zone shows a higher β2-AR expression in hanging, fire fatalities, and road accidents; (d) medulla expression was greater in cases of hanging and drowning (Figure 3).

## 3. Discussion

The aim of this study was to analyze the structural and/or molecular changes in adrenal glands obtained from forensic autopsies of different causes of death, in order to evaluate the presence of β2-AR in the four zones of the glands and to investigate its expression in subjects that died during different stressful conditions. In particular, the study was directed to analyze an adrenal tissue marker involved in the stress response process to activated/stimulated receptors by the activation of the SA system and HPA axis, focalizing the attention exclusively on the adrenal gland and its specific functions.

The study showed that β2-ARs were expressed in the human medulla and cortex and that cortical expression was observed in all zones, suggesting that in the stress response, the adrenergic system could contribute, as a complementary pathway, to stimulate steroid production and release involving the glomerulosa, the fasciculata and the reticularis, respectively secreting mineralocorticoids, glucocorticoids and androgens [24]. While the involvement of glucocorticoids and catecholamines in the stress response is well described, few studies examine the hormones secreted from the glomerulosa and reticularis, which are regulated by adrenocorticotropic hormone (ACTH) [25]. Therefore, to the best of our knowledge, the present study is the first:

(i) to histologically demonstrate β2-AR expression in the human cortex;
(ii) to provide suggestions on the possible involvement of β2-AR in human cortex hormonal stimulation, and
(iii) to study β2-AR expression in the adrenal gland in different causes of death.

The presence of β2-AR in the cortical zone of the human adrenal gland might indicate that the cortex and medulla, even if embryologically different, could share common mechanisms in the coordination of the stress response.

As to the possible involvement of β2-AR in human cortex hormonal stimulation, the results have demonstrated that an in vivo cross-talk between cortical and medullary cells was present according to previous evidence based on vascular and nervous adrenal features. Adrenomedullary cells receive blood from the cortex in which high concentrations of adrenocortical steroids are present [3] and cortical nerves derived from the sympathoadrenal cell lineage are organized in an intricate plexus in the subcapsular region which extends into the fasciculata and reticularis layers [26,27,28,29]. Thus, the reported findings confirm the importance of medullary–cortical interactions and the role of the sympathoadrenal system and catecholamines when stress occurs. The presence of β2-ARs in all the cellular layers of the adrenal cortex represents the morphological basis to explain the existence (in addition to ACTH release) of paracrine regulation of adrenocortical steroidogenesis from medullary cells [30]. In fact, it was observed that the perfusion of the isolated porcine adrenal glands with epinephrine or norepinephrine induced the secretion of cortisol, aldosterone, and androstenedione [30]. Therefore, the results of the immunohistochemical localization of β2-ARs could represent the morphological prerequisites for an intra-adrenal regulatory interaction during the stress response.

Moreover, the study showed differing β2-AR expression, for both localization and quantity, in the adrenal glands of subjects who died from different causes, suggesting that the activation/stimulation of adrenergic receptors might vary on the basis of different stressful conditions related to the death process.

Although many studies have highlighted the increase of catecholamines and glucocorticoids in relation to stress [3,6], the findings of our study documented the involvement of the whole gland: the glomerulosa and reticularis were also stimulated together with the fasciculata and medulla layers. It was interesting to discover that in some causes of death, the expression of the receptors appeared predominant in these other two zones; in fact, only sudden cardiac death and hanging showed a greater expression of β2-AR respectively in the fasciculata and medulla.

In the sudden cardiac death group, the greater expression in the fasciculata suggested that the adrenal gland response could be mainly associated with the synthesis and secretion of glucocorticoids, possibly related to their effect on blood pressure regulation and on evidence suggesting the involvement in heart rate control by the arterial baroreceptor reflex [31,32,33].

In the hanging group, the asphyxia condition could induce a greater stimulation of medullary β2-ARs to promote the synthesis and secretion of catecholamines as supported by evidence on adrenalin and noradrenaline increase due to both blood pH alteration induced by hypoxia and activation of a sympathetic–adrenal mechanism regulating the cardiovascular response to asphyxia (redistribution of blood flow toward the brain and muscle beds). In previous experimental studies on the analysis of the effects of asphyxia, an increase of systemic arterial pressure, sympathetic vertebral nerve activity, nitric oxide formation and the release of plasma catecholamine components of norepinephrine and epinephrine was observed [34,35]; moreover post-mortem biochemical analysis on blood from subjects that died of asphyxiation revealed high levels of adrenalin [7]. The coexistence of β2-AR overexpression in the glomerulosa and reticularis could be related to the described neuroprotective effects of mineralcorticoids and dehydroepiandrosterone (DHEA), considering that the mechanisms of death in hanging are generally vagal inhibition and/or asphyxia and/or cerebral ischemia and/or cerebral congestion [36]. Some researchers have shown an increase in mineralcorticoid receptors in specific cerebral areas (i.e., the hippocampus) after asphyxia, supporting the substantial role of corticoids in the neural response to conditions of oxidative stress and ischemia/hypoxia, even if the mediated neuronal mechanism remains unclear [37]. The neurotrophic and/or neuroprotective activity in the central nervous system was observed also for DHEA on the basis of strong evidence, suggesting its effects on GABAergic transmission modulation in the central nervous system [38,39].

In the road accident group, including subjects with brain injuries, the effects of DHEA and DHEA sulfate (DHEAS) could explain the higher β2-AR expression observed in the reticularis zone, as these hormones have positive effects in brain lesions as documented by experimental studies [40,41].

In the fire fatality group, the significant β2-AR expression in the reticularis could be related to the modulation of the nociceptive function of DHEA and DHEAS [42,43], in particular in three of our subjects who died quickly from severe burns and, possibly, in intense painful conditions.

In the sepsis group, the higher β2-AR expression in the glomerulosa suggested a role for mineralocorticoid synthesis and secretion, related to compensatory mechanisms activated to increase volemia and arterial blood pressure through the renin–angiotensin–aldosterone system (RAAS) [44,45]. It is well known that the activation of the RAAS is part of an intrinsic neuroendocrine response to sepsis-related circulatory failure also mediated by the activation of the hypothalamic–pituitary–adrenal axis [46,47].

In the drowning group, the higher expression of β2-AR in the glomerulosa is possibly related to the systemic effects of drowning fluid. Studies on pathophysiology highlighted that drowning determines different systemic responses and outcomes, in relation to immersion (upper airway above water) and/or submersion (upper airway under water), depending also on the characteristics of the drowning liquid (i.e., temperature and osmolarity). These systemic effects affect the cardiocirculatory, respiratory and neural systems by several mechanisms, mediated by hypothermia, hypovolemia, diving response, autonomic conflict, upper airway reflexes, water aspiration and swallowing, and electrolyte disorders [48,49]. In light of this, on the basis of the results of the present study, it could be supposed that there is a role for mineralcorticoids in reacting to drowning-related phenomena to cause hypovolemia and electrolyte modifications.

Therefore, the present study highlights the post-mortem profile of β2-AR expression in the human adrenal gland, showing topographic and quantitative differences, possibly related to different causes of death. These results suggest that other studies aiming to evaluate the correlation between adrenal β2-AR expression and the activation of SA system and HPA axis (not investigated in the present study) could be very useful to improve the knowledge of stress response processes. Furthermore, in a medicolegal approach to the causes and mechanisms of death, adrenal gland analysis could prove to be, as well as other second level investigations [50,51,52,53,54,55,56], a useful tool to improve the etiopathogenetic assessment of stress-related death.

The major limits of our study are represented by the relatively small number of samples and by the possible influence on β2-ARs expression caused by the subject’s previous pharmacological therapy (data not known). Furthermore, post-mortem changes cannot be evaluated in this study, thus no comments can be offered on the effects of post-mortem phenomena on receptor expression. The authors did not analyze the relationship between the obtained results and other factors (age and sex, agony duration), which would also possibly affect the expression of the receptors.

## 4. Materials and Methods

Forty-eight forensic autopsies, carried out between March 2017 and December 2017, were included in this study (Table 1). The following causes of death were included: (i) hanging (*n* = 8; 3 females and 5 males; mean age 51.4 ± 17 years); (ii) road accident (*n* = 10; 6 women and 4 men; mean age 56.3 ± 23.6 years); (iii) fire fatality (*n* = 4; 1 woman and 3 men; mean age 46.3 ± 22.9); (iv) sudden cardiac death (*n* = 8; 2 women and 6 men; mean age 52.7 ± 16.9 years); (v) sepsis (*n* = 8, 6 women and 2 men; mean age 67.4 ± 10.7 years); and (vi) drowning (*n* = 10; 3 women and 7 men; mean age 28.5 ± 15.3 years). All autopsies were performed 24–48 h after death.

This is a retrospective study carried out on filed judiciary cases in which the specimens were taken during autopsy as ordered by a judicial authority. The authors asked for ethical approval (prot. n. 24/19, 28/2/2019) from the Local Ethics Committee (Comitato Etico Interaziendale della Provincia di Messina) that declared no-competence because the study used judiciary specimens. The authors got the permission to use the autopsy samples for research purposes by the General Public Prosecutor.

### 4.1. Immunohistochemistry Analysis

Adrenal glands obtained from the above autopsies were fixed in 4% paraformaldehyde in 0.2 M phosphate buffered saline (PBS), dehydrated in graded ethanol, cleared in xylene and embedded in paraffin (Paraplast, SPI Supplies, West Chester, PA, USA). The histological sections (5 μm) were deparaffinized in xylene and rehydrated in ethanol. Antigen retrieval was performed with pH 6.0 citrate buffer and endogenous peroxidase blocked with 0.3 % H_2_O_2_ in PBS. The primary antibody (β2-AR, Santa Cruz, Dallas, TX, USA; 1/100 dilution) was incubated overnight at 4 °C in a moisturized chamber. The following day, peroxidase-conjugated secondary antibody (Pierce anti-rabbit, anti-goat, and anti-mouse, Cambridge, UK; 1/50 dilution) was added and the reaction was visualized with 3,3′-diaminobenzidine (DAB) (Sigma-Aldrich, Milan, Italy). Counterstaining was performed in Mayer’s hematoxylin (alum hematoxylin). Negative controls from all the regions of the adrenal gland (glomerulosa, fasciculata, reticularis and medulla) were obtained using PBS instead of the primary antibody (supplementary materials). For the positive control, normal human pancreas sections were used according to the manufacturer’s instructions.

### 4.2. Morphometric Analysis

The sections were photographed with a Nikon Ci-L (Nikon Instruments, Tokyo, Japan) light microscope. All images were taken with a digital camera (Nikon Ds-Ri2) and saved as Tagged Image Format Files (TIFF) with the Adobe Photoshop CS software. Five randomly chosen microscopic fields (MFs) from five non-serial sections of each group were considered. All micrographs were captured at the same magnification and were blindly assessed by two trained observers who ignored which of the groups the examined specimens belonged to. Immunopositivity of β2-AR in each zone of the adrenal gland (glomerulosa, fasciculata, reticularis and medulla) was graded according to the following semi-quantitative score: 0, negative; 1, slight and/or limited (to a few groups of cells) positivity; 2, patchy and scattered positivity; 3, diffuse and intense positivity.

### 4.3. Statistical Analysis

For each cause of death, data referring to the semi-quantitative grading of β2-AR expression assigned to the different zones of all glands were expressed as mean ± standard deviation (SD). A non-parametric approach was used since the examined variables were not normally distributed, as verified by the Kolmogorov–Smirnov test. For each cause of death, a non-parametric ANOVA was applied in order to assess the existence of possible differences among the four adrenal gland zones (intra-groups analysis). The same analysis was performed in order to assess, for each zone, possible differences among the six causes of death (inter-groups analysis). Statistical analysis was performed using the SPSS 17.0 for Window package. A *p*-value of ≤0.05 was considered statistically significant.

## 5. Conclusions

This study revealed the expression of β2-ARs in all layers of the human adrenal gland and supports the presence of a cross-talk between cortical and medullary cells.

The obtained results suggest, also, that the adrenal gland could respond to stress with a kind of “specificity”, managing homeostatic changes determined by different stressors and stressful conditions, possibly providing a specific response in different death processes.

The authors state that, considering the poor literature about adrenal gland β2-ARs and the absence of previous studies focused on adrenal gland functional changes during death processes, the obtained results must be considered as preliminary evidence supporting the opportunity to perform further research aimed to improve the knowledge of the adrenal gland’s functionalities and to discover its role in human pathophysiology during stress conditions.

## Figures and Tables

**Table 1:** Age, sex, cause of death, adrenal cortical scores and adrenal medullary scores in the case series.

					Cortex		Medulla
Case	Age	Sex	Cause of Death	Glomerulosa	Fasciculata	Reticularis	
1	53	F	Hanging	3	1	3	3
2	44	F		3	1	3	3
3	57	F		2	1	2	2
4	38	M		3	0	2	3
5	66	M		3	1	3	2
6	70	M		2	0	2	2
7	64	M		3	1	3	3
8	19	M		2	1	3	2
9	79	M	Road accident	0	1	2	1
10	73	M		0	1	2	1
11	23	M		1	0	3	1
12	30	F		0	0	2	0
13	66	F		0	1	1	1
14	58	F		1	1	2	0
15	19	M		1	1	3	1
16	80	F		0	0	2	1
17	75	F		0	1	3	1
18	60	F		0	1	2	1
19	19	M	Fire fatality	0	2	3	0
20	52	M		0	2	3	0
21	74	M		1	2	2	1
22	40	F		0	1	3	0
23	33	F	Sudden death	1	3	0	1
24	27	M		1	3	0	1
25	61	M		0	2	0	0
26	63	F		1	2	1	1
27	68	M		0	3	0	0
28	56	M		1	2	0	1
29	41	M		1	3	0	0
30	73	M		1	3	0	1
31	67	F	Sepsis	2	0	1	0
32	82	F		2	0	1	0
33	62	M		1	0	1	0
34	72	F		3	0	1	1
35	74	M		2	0	0	0
36	54	F		3	1	2	1
37	76	F		2	0	1	0
38	52	F		2	1	1	0
39	18	M	Drowning	3	1	1	2
40	21	M		2	0	0	1
41	18	M		3	0	1	2
42	23	F		2	1	1	1
43	58	M		3	0	1	2
44	53	F		2	1	0	2
45	18	F		3	0	1	1
46	22	M		2	1	0	2
47	18	M		2	0	0	1
48	36	M		3	1	0	1

## Supplementary Materials

The supplementary materials are available online at https://www.mdpi.com/1422-0067/20/12/3065/s1. Representative images of positive and negative controls are reported in the supplementary materials.
