# Supplementary material for: Post-Mortem Immunohistochemical Evidence of β2-Adrenergic Receptor Expression in the Adrenal Gland
Source: Int J Mol Sci. 2019 Jun 23;20(12):3065. doi: 10.3390/ijms20123065 (PMC6628614; doi:10.3390/ijms20123065)

Supplementary material

INTERNATIONAL JOURNAL OF MOLECULAR SCIENCES

Post-mortem immunohistochemical evidence of β2-Adrenergic receptor expression in the adrenal gland

Elvira Ventura Spagnolo [1,*], Cristina Mondello [2,*], Luigi Cardia [3], Letteria Minutoli [4], Domenico Puzzolo [2], Alessio Asmundo[2], Vincenzo Macaione [4], Angela Alibrandi [5], Consuelo Malta [2], Gennaro Baldino[1] and Antonio Micali [2]

[1] Legal Medicine Section, Department for Health Promotion and Mother-Child Care, University of Palermo, Via del Vespro, 129, 90127, Palermo, Italy
[2] Department of Biomedical and Dental Sciences and Morphofunctional Imaging, University of Messina, via Consolare Valeria, 1, 98125 Messina, Italy
[3] Department of Human Pathology of Adult and Childhood "Gaetano Barresi", University of Messina, Via Consolare Valeria, 98125, Gazzi, Italy
[4] Department of Clinical and Experimental Medicine, University of Messina, via Consolare Valeria, 1, 98125 Messina, Italy
[5] Department of Economics, Unit of Statistical and Mathematical Sciences, University of Messina, Via dei Verdi 75, 98122 Messina, Italy

* Corresponding author: Elvira VENTURA SPAGNOLO - elvira.ventura@unipa.it; Cristina MONDELLO – mondelloc@unime.it

Figure 1. Representative image of normal human pancreas section used as positive control.

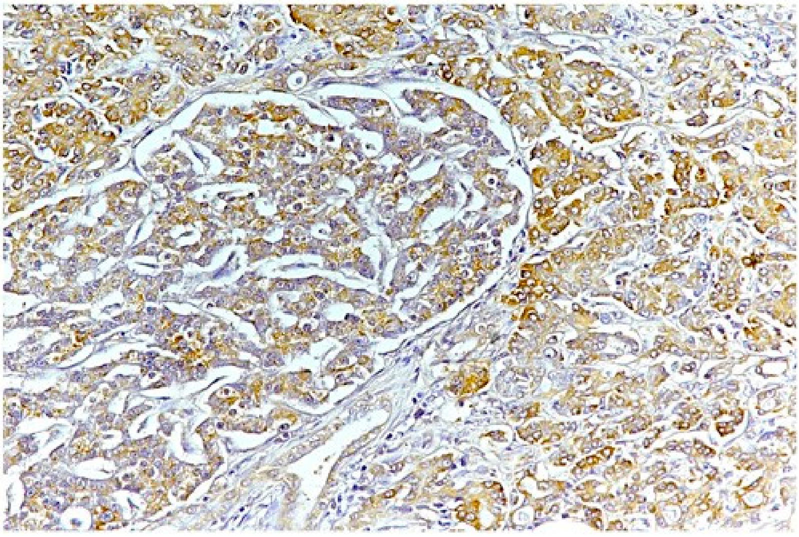

Figure 2. Representative panel of negative controls obtained, using PBS, from all the regions of the adrenal gland (glomerulosa, fasciculata, reticularis and medulla).

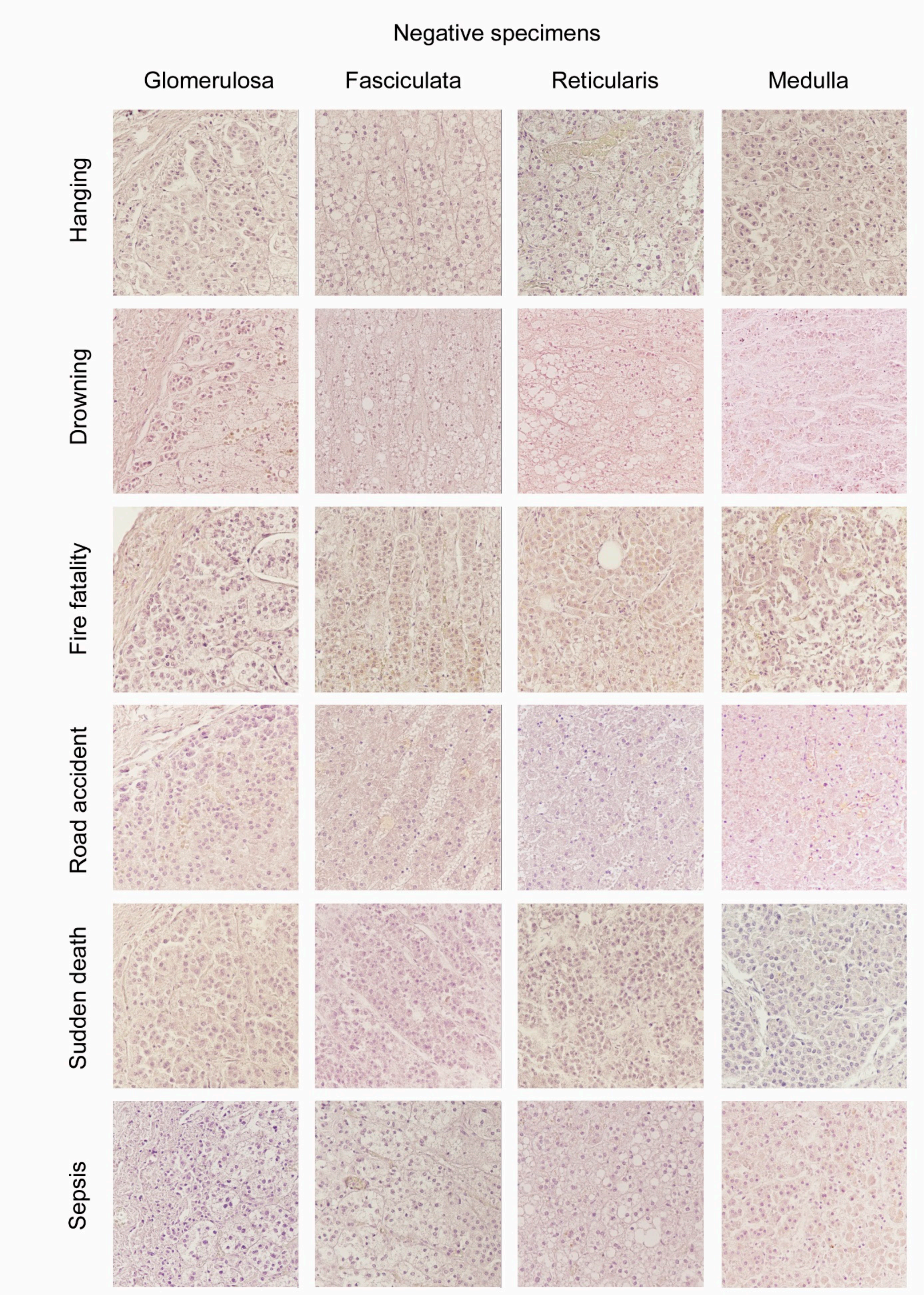

Supplement: Supplementary file 1 [file ijms-20-03065-s001.pdf]
